# Hypertrophic Obstructive Cardiomyopathy: Comparison of Outcomes After Myectomy or Alcohol Ablation

**DOI:** 10.3389/fcvm.2022.755376

**Published:** 2022-03-14

**Authors:** Xiangbin Meng, Wen-yao Wang, Jun Gao, Kuo Zhang, Jilin Zheng, Jing-jia Wang, YuPeng Liu, Chunli Shao, Yi-Da Tang

**Affiliations:** ^1^Key Laboratory of Molecular Cardiovascular Science, Ministry of Education, Department of Cardiology and Institute of Vascular Medicine, Peking University Third Hospital, Beijing, China; ^2^State Key Laboratory of Cardiovascular Disease, Department of Cardiology, National Center for Cardiovascular Diseases, Fuwai Hospital, Chinese Academy of Medical Sciences and Peking Union Medical College, Beijing, China

**Keywords:** hypertrophic obstructive cardiomyopathy, myectomy, alcohol septal ablation, prognosis, LVOT

## Abstract

**Introduction and Objectives:**

The risk of ventricular arrhythmia and heart failure in patients with hypertrophic obstructive cardiomyopathy (HOCM) is much higher than that in the general population. More and more pieces of evidence showed that HOCM is the leading cause of sudden cardiac death in young people. We reported our experience in a study, comparing surgical myectomy, alcohol septal ablation (ASA), and medical therapy.

**Methods:**

The original cohort included 965 consecutive patients with HOCM. The patients were divided into three groups according to treatment strategies: myectomy group (*n* = 502), ASA group (*n* = 138), and medical treatment group (*n* = 325). The median follow-up duration was 42.99 ± 18.32 months, and the primary endpoints were all-cause mortality and heart transplantation.

**Results:**

Both in short- and long-term observations, surgical myectomy reduced the left ventricular outflow tract (LVOT) gradients more effectively (7 days, 16.15 ± 12.07 mmHg vs. 42.33 ± 27.76 mmHg, *p* < 0.05; 1 year, 14.65 ± 13.18 mmHg vs. 41.17 ± 30.76 mmHg, *p* < 0.05). Among the three groups, the patients in the medical treatment group were at a higher risk of mortality and cardiac transplantation (vs. the myectomy group, *p* < 0.001 by log-rank test; vs. the alcohol septal ablation group, *p* = 0.017 by log-rank test), and the myectomy group shows a lower risk of reaching the primary endpoint than the two other groups. In the multivariate Cox regression analysis, previous atrial fibrillation (AF), N terminal pro B type natriuretic peptide (NT-pro-BNP), and surgical myectomy predicted an HOCM prognosis. However, the impact of surgical myectomy on HOCM prognosis seems to be limited to the <56 years group.

**Conclusions:**

The patients with medical treatments seemed to suffer from the highest risk of achieving an all-cause mortality and the endpoint of heart transplantation. In the long-term survival and clinical outcome, myectomy seemed better than alcohol septal ablation, especially the younger patients. Due to the less-controllable degree, periprocedural complication frequency after alcohol septal ablation was higher, compared with myectomy. Furthermore, gradients after myectomy are lower at late follow-up. To sum up, when selecting treatment strategies, the patients should be individually evaluated by a multidisciplinary team of cardiologists and surgeons.

## Keypoints

### What Is Known About the Topic?

Hypertrophic obstructive cardiomyopathy is common cardiomyopathy. The relief of left ventricular outflow tract obstruction is the main method of an intervention treatment. Although surgical treatment is considered to be the “gold standard” of treatment, there is still a lack of randomized controlled trials to analyze the long-term therapeutic effect of surgical treatment and ablation. Our research aims to provide more clinical evidence in this field.

### What Does This Study Add?

This study adds to the increasing evidence that myectomy seemed better than alcohol septal ablation, especially in the younger patients. After ASA, the periprocedural complication frequency was higher, reflecting its less-controllable degree invasive nature compared with myectomy. On the other hand, gradients after myectomy are lower at late follow-up, which could favor myectomy.

## Introduction

Hypertrophic cardiomyopathy (HCM) is genetic heart disease. Its features are characterized by cardiomyocyte hypertrophy, disarray, interstitial fibrosis, and ventricular hypertrophy, mainly involving an inter-ventricular septum and left ventricle (1). Compared with the average population, patients with HCM have a higher risk of ventricular arrhythmia and heart failure. More and more pieces of evidence showed that HCM is the leading cause of sudden cardiac death in young people (especially under 25 years) (2–4). About 70% of patients with HCM are classified as hypertrophic obstructive cardiomyopathy (HOCM). The HOCM is always accompanied by left ventricular outflow tract (LVOT) blood flow acceleration (5). Although medical treatment can provide relief of symptoms, a significant subset of patients with HOCM still has symptoms. Among these subjects, invasive treatment is an established treatment strategy (6, 7). Both surgical myectomy and alcohol septal ablation (ASA) have been proven effective in alleviating the symptoms (8–10). In recent studies, ASA has been associated with good survival, comparable to age- and sex-matched populations (11, 12). Since ASA is also a less-invasive treatment, it may be a preferred treatment.

On the other hand, previous studies have also reported that the necessity of pacemaker implantation is more frequent after the ASA compared with myectomy (7, 8). The ASA may also increase the cardiac mortality in a single-center study (13). We reported the consequences of two invasive HOCM treatments, including perioperative complications, survival rate, cardiac survival rate, long-term symptom status, and clinical results.

## Methods

### Ethics Statement

The study followed the ethical guidelines of the declaration of Helsinki and of China's regulations and policies on good clinical practice and was approved by the Ethics Committee of XXXX1. Before the start of the study, we had obtained written informed consent from all the participants.

### Study Patients

All the patients in this study were evaluated in the hospital of XXXX1. Between October 1, 2009 and December 31, 2014, a total of 965 patients (age ≥ 16 years), diagnosed with HOCM, were included. All clinical and medical data were available. Any cardiac or systemic diseases that could produce significant hypertrophy were excluded, such as uncontrolled hypertension (family blood pressure monitoring) ≥ 140/90 mmHg), congenital heart disease, heart valve disease, and amyloidosis. Among those patients, 502 patients accepted the surgical myectomy, and 138 patients got the alcohol septal ablation. At the same time, 325 patients did not receive invasive treatment strategies for various reasons but only chose drug treatment. The diagnosis of HOCM based on (14, 15) the following: (1) wall thickness ≥15 mm in one or more LV myocardial segments measured by any imaging technique (echocardiography, cardiac magnetic resonance imaging, or computed tomography); (2) wall thickness (13–14 mm) with family history, ECG abnormalities, non-cardiac symptoms and signs, laboratory tests, and multi-modality cardiac imaging; and (3) LVOT obstruction (LVOTO) based on: the dynamic LVOT block due to systolic anterior motion of the mitral valve, with an LVOT gradient ≥30 mmHg at rest or during physiological provocation such as Valsalva maneuver, standing, and exercise. The LVOT obstruction was distinguished mainly by two-dimensional and Doppler echocardiography.

Invasive treatment should be considered in patients with an LVOTO gradient of ≥50 mmHg resting or with provocation, moderate to severe symptoms (NYHA III–IV), and/or recurrent exertional syncope after maximally trying a tolerated drug treatment. After discussing the risks and benefits of each strategy, alcohol septal ablation or surgical myectomy was selected through a standard decision-making process. Surgical myectomy is considered as the gold standard therapy for HOCM. Excision of protruding septal muscle results in enlargement of the left ventricular outflow tract, with a decrease in the severity or complete elimination of the left ventricular outflow tract obstruction (16). Transcatheter alcohol septal ablation is another preferred alternative treatment. Alcohol is injected directly into a septal branch of the anterior-descending artery supplying the basal part of the septum through an interventional catheter to induce a local myocardial necrosis, to lead to scar formation, and to reduce left ventricular outflow tract obstruction (17).

### Follow-Up and Endpoints

The follow-up began when the patient has first contacted the clinic after October 1, 2009. At the baseline, all the patients were assessed for the following characteristics: age, gender, NYHA functional grade, maximum left ventricular wall thickness (LVWT), maximum provocation of LVOT gradient, left ventricular function, risk factors for atrial fibrillation, and sudden cardiac death (SCD). Perioperative adverse events such as implantable cardioverter-defibrillator/pacemaker were also collected.

The primary endpoints of this study were all-cause mortality and heart transplantation during the long-term follow-up. Mortality and adverse events were retrieved from the hospital patient records at the center where follow-up occurred, from civil service population registers, and from the information provided by the patients themselves and/or their general practitioners. The patients lost to follow-up were censored upon the last contact with them. If no events occurred during follow-up, the administrative censoring date was set at December 31, 2016.

### Data Analysis

Statistical analysis was assessed with SPSS 21.0 statistical package for Windows. All continuous variables were presented as means ± SD, and analysis of variance was used to compare means across multiple groups. The relationships between parametric variables were assessed by multiple linear regression analysis. Initial differences in baseline characteristics between achieved treatment groups were sought in bivariable analysis using χ^2^ tests, Fisher exact tests, Student *T-*tests, and Kruskal–Wallis analysis of variance. Cox, the proportional hazards model, was used to estimate the hazard ratio (HR). Kaplan–Meier analysis was used to study the cumulative survival of different groups. A *P*-value < 0.05 was considered statistically significant.

## Results

### Study Population and Baseline Clinical Characteristics

From October 1, 2009, to December 31, 2014, 965 consecutive patients with HOCM (age ≥ 16 years) were admitted to our center. About 138 patients received ASA, 502 patients underwent myectomy, and 325 patients did not receive invasive treatment. Baseline characteristics of the three treatment groups are shown in Table 1. According to the treatment strategy, the population was divided into three groups. The patients in the myectomy group had the highest NT-proBNP level and the creatinine level, the lowest systolic/diastolic BP level, and the lowest percentage of atrial fibrillation or non-sustained ventricular tachycardia and the rate of coronary artery disease. Patients in the medical treatment group being older, having a higher blood pressure value and a more rate of hypertension, dyslipidemia and arrhythmia.

**Table 1:** Baseline clinical characteristics.

	**Total**	Myectomy^^*^^	**ASA**	**Medical treatment**	***P*-value**
	**(*n =* 965)**	**(*n =* 502)**	**(*n =* 138)**	**(*n =* 325)**	
**Demographics**					
Age, y	50.31 ± 12.84	47.22 ± 11.85	50.39 ± 10.60	55.05 ± 13.72	A; a; b; c
Male, *n* (%)	580 (60.1%)	301 (60.0%)	90 (65.2%)	189 (58.2%)	NS
BMI (kg/m^2^)	25.56 ± 5.45	25.24 ± 4.09	26.00 ± 3.14	25.92 ± 7.77	NS
Smoking, *n* (%)	417 (43.2%)	205 (40.8%)	65 (47.1%)	147 (45.2%)	NS
Systolic BP (mmHg)	119.54 ± 18.15	115.6 ± 16.26	118.55 ± 15.73	126.06 ± 19.99	A; a; b; c;
Diastolic BP (mmHg)	73.1 ± 11.30	71.02 ± 10.93	73.44 ± 11.06	76.17 ± 11.30	A; a; b; c;
NT-proBNP (fmol/mL)	1,881.20 ± 1,701.50	2,118.59 ± 1,882.52	1,530.32 ± 1,100.98	1,729.05 ± 1,640.43	A; a; b;
Cr (μmol/L)	78.37 ± 27.28	80.30 ± 9.97	76.00 ± 18.42	76.18 ± 20.84	A; b;
**Comorbidities and risk factors**					
Hypertension, *n* (%)	293 (30.4%)	98 (19.5%)	44 (31.9%)	151 (46.5%)	A;a;b;
Diabetes mellitus, *n* (%)	61 (6.3%)	17 (3.4%)	17 (12.3%)	27 (8.3%)	A;a;b;c
Dyslipidemia, *n* (%)	234 (24.2%)	68 (13.5%)	35 (25.4%)	131 (40.3%)	A;a;b;
Atrial fibrillation, *n* (%)	123 (12.7%)	51 (10.2%)	15 (10.9%)	57 (17.5%)	A;a;b;c;
Non-sustained ventricular tachycardia, *n* (%)	24 (2.5%)	4 (0.8%)	4 (2.9%)	16 (4.9%)	A;a;b;c;
Coronary artery disease	176 (18.3%)	69 (13.8%)	20 (14.6%)	87 (26.8%)	A,b,c
Clearly family history of HCM, *n* (%)	131 (13.6%)	83 (16.5%)	9 (6.5%)	39 (12.0%)	NS
NYHA Class III or IV, *n* (%)	83 (11.0%)	40 (17.5%)	21 (8.1%)	22 (8.1%)	NS
**Echocardiography**					
Interventricular septal thickness (mm)	20.00 ± 5.42	19.73 ± 5.32	21.09 ± 4.90	19.97 ± 5.74	A; a;
LV end-diastolic diameter (mm)	42.50 ± 6.12	42.74 ± 5.70	41.45 ± 5.75	42.57 ± 6.83	A; a; c;
LV posterior wall thickness (mm)	11.99 ± 2.89	12.04 ± 2.80	12.26 ± 2.77	11.79 ± 3.08	NS
LV ejection fraction (%)	67.79 ± 8.88	66.71 ± 8.48	70.84 ± 7.90	68.15 ± 9.55	A; a; c;
LV outflow tract gradient, at rest (mmHg)	73.97 ± 34.37	77.10 ± 34.05	89.36 ± 31.03	63.13 ± 32.91	A; a; b; c;
LV outflow tract gradient, during physiological provocation	86.91 ± 39.42	88.37 ± 37.73	89.36 ± 31.04	82.64 ± 45.90	NS
LV hypertrophy ≥30 mm, *n* (%)	60 (6.2%)	30 (6.0%)	6 (4.3%)	24 (7.4%)	NS
**Medications**					
Beta-blocker, *n* (%)	482 (49.9%)	241 (48.0%)	65 (47.1%)	176 (54.2%)	A; a; c;
ACEI/ARB, *n* (%)	78 (8.1%)	14 (2.8%)	16 (11.6%)	48 (14.8%)	A; a; b; c;
Statin, *n* (%)	116 (12.0%)	26 (5.2%)	18 (13.0%)	72 (22.2%)	A; b; c
Calcium antagonist, *n* (%)	136 (14.1%)	32 (6.4%)	17 (12.3%)	87 (26.8%)	A; b; c;
**Additional intervention**					
Myectomy + CABG	–	44 (8.8%)	–	–	–
ASA + PCI	–	–	9 (6.5%)		

*Values are mean ± SD or n (%)*.

*BMI, body mass index; NT-proBNP, N-terminal pro-brain natriuretic peptide; Cr, serum creatinine; BP, blood pressure; NYHA, New York Heart Association; LV, left ventricle; ASA, alcohol septal ablation; CABG, coronary artery bypass grafting; PCI, percutaneous coronary intervention; ACEI/ARB, angiotensin-converting enzyme inhibitor/angiotensin receptor blocker*.

*“NS”: not significant; “A”: Significant difference between three groups; “a”: Significant difference between the Myectomy group and ASA group. “b”: Significant difference between the Myectomy group and “Without Invasive treatment” group. “c”: Significant difference between the ASA group and “Without Invasive treatment” group*.

* *including 8 patients had resaved alcohol septal ablation before septal myectomy (They all in myectomy group)*.

Myectomy combined with CABG was more common than ASA combined with percutaneous coronary intervention. In patients with ASA, interventricular septal thickness, left ventricular ejection fraction, and left ventricular outflow tract gradient were slightly more significant than those in the other two groups. There were significant differences in age, history of hypertension, diabetes, dyslipidemia, and medication history between the three groups. In the three groups, no significant difference was found in the percentage of men/smoking/family history/BMI level/NYHA class. In addition, eight patients had received an alcohol septal ablation before myectomy (all in the myectomy group).

### Periprocedural Complications and LVOT Gradient Changes After Invasive Treatment

The patients in myectomy group had a lower LVOT gradient in 7 days/1 year after the invasive treatment, and a lower percentage of ventricular fibrillation/sustained VT, and a lower percentage of implantable cardioverter-defibrillator (ICD)/Pacemaker (16.15 ± 12.07 mmHg vs. 42.33 ± 27.76 mmHg, *p* < 0.05; 14.65 ± 13.18 mmHg vs. 41.17 ± 30.76 mmHg, *p* < 0.05; 0.8% vs. 2.9%, *P* = 0.049; 0.4% vs. 17.4%, *p* < 0.05). Three patients in the myectomy group died after surgical myectomy during the perioperative period (30 days) (Table 2).

**Table 2:** Periprocedural complications (30 days) and LVOT gradient changes after invasive treatment.

	**Myectomy** **(*n =* 502)**	**ASA** **(*n =* 138)**	***p*-value**
LVOT gradient (7 days after Invasive treatment, mmHg)	16.15 ± 12.07	42.33 ± 27.76	<0.05
LVOT gradient (1 year after Invasive treatment, mmHg)	14.65 ± 13.18	41.17 ± 30.76	<0.05
Periprocedural death	3 (0.6%)	0 (0)	0.363
Ventricular fibrillation/sustained VT	4 (0.8%)	4 (2.9%)	0.049
Implantable cardioverter defibrillator/ Pacemaker	2 (0.4%)	24 (17.4%)	<0.05

### Survival and Clinical Outcome

**Figure 1:**
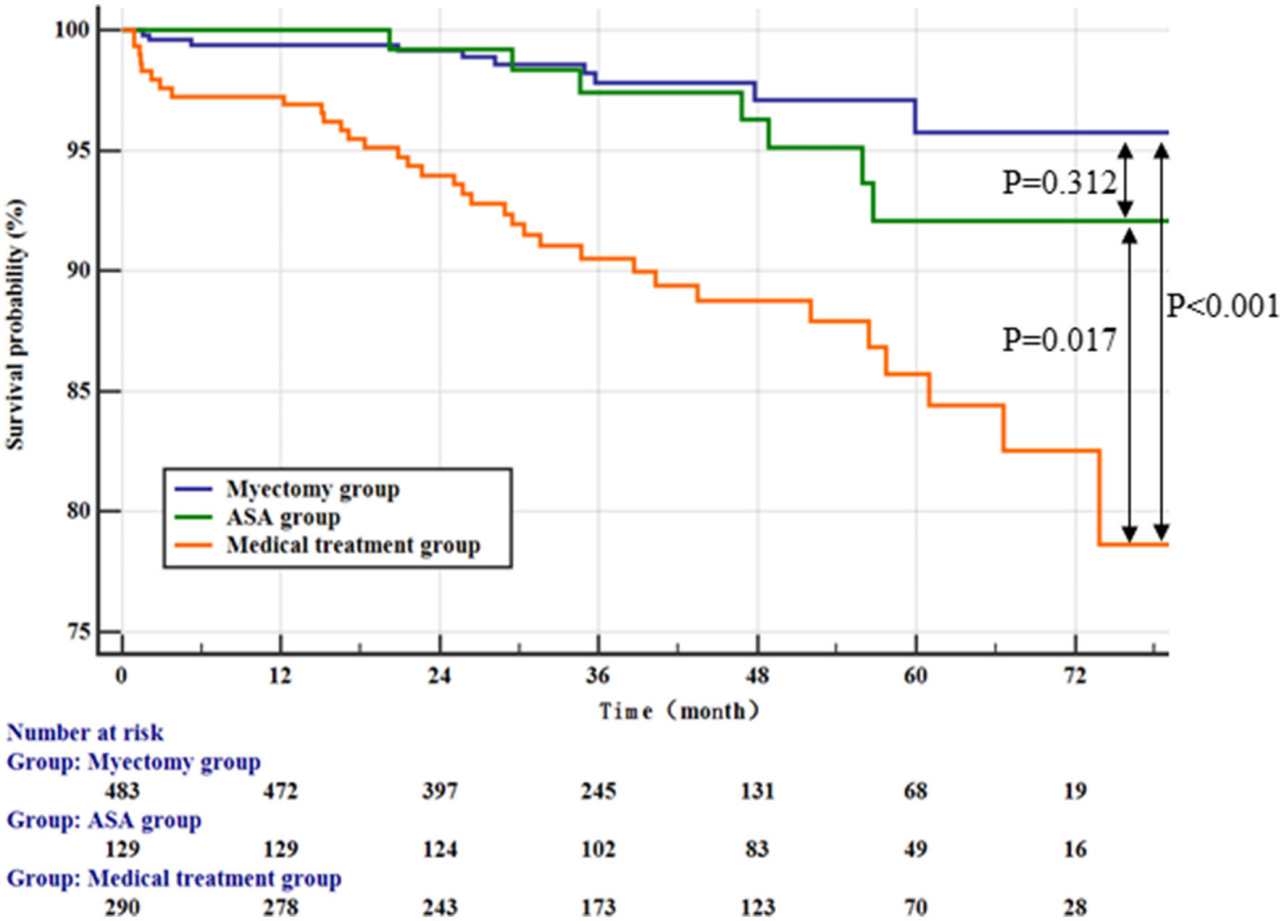
Kaplan–Meier curves comparing the probability of outcomes stratified by treatment strategies.

After a median follow-up of 42.99 ± 18.32 months, 49 patients (5.1%) died, with rates of 1.8%, 5.1%, and 10.2% in the myectomy group, ASA group, and medical treatment group, respectively. In addition, two patients with end-stage heart failure underwent orthotopic heart transplantation, 1 (0.2%) from the myotomy group and 1 (0.8%) from the ASA group (Table 3). Among the three groups, the patients in the medical treatment group suffered the highest risk of reaching the all-cause mortality and cardiac transplantation endpoint (Figure 1; vs. the myectomy group, *p* < 0.001 by log-rank test; vs. the ASA group, *p* = 0.017 by log-rank test). The myectomy group showed the lowest risk of reaching the endpoint than the other two groups, but there was no statistical difference compared with ASA (Figure 1; vs. the medical treatment group, *p* < 0.001 by log-rank test; vs. the ASA group, *p* = 0.312 by log-rank test).

**Table 3:** Clinical outcome.

	**Total**	**Myectomy**	**ASA**	**Medical treatment**	***p*-value**
	**(*n =* 965)**	**(*n =* 502)**	**(*n =* 138)**	**(*n =* 325)**	
Follow-up duration, Mos.	42.99 ± 18.32	39.22 ± 16.46	53.57 ± 16.93	44.31 ± 19.67	A; b; c;
Cardiac transplantation	2 (0.2%)	1 (0.2%)	1 (0.8%)	0 (0)	NS
All-Cause Mortality	49 (5.1%)	9 (1.8%)	7 (5.1%)	33 (10.2%)	A; a; b; c;
All-Cause Mortality and Cardiac transplantation	51 (5.3%)	10 (2%)	8 (5.8%)	33 (10.2%)	A; a; b; c;

*“NS”: not significant; “A”: Significant difference between three groups; “a”: Significant difference between the Myectomy group and ASA group. “b”: Significant difference between the Myectomy group and “Without Invasive treatment” group. “c”: Significant difference between the ASA group and “Without Invasive treatment” group (Conservative treatment group)*.

Table 4 shows the univariate cox regression analysis results of all-cause mortality and the cardiac transplantation endpoint. Age, previous AF, LVEF, LV end-diastolic diameter, Creatinine, and treatment strategy (ASA and myectomy) showed a significant predicting value on HOCM prognosis. In multivariate cox regression analysis, age, previous AF, Creatinine, LVEF, and myectomy treatment strategy, still, have significant predicting value on HOCM prognosis (for age, HR: 1.051; 95% CI: 1.022–1.080, *p* < 0.001; for previous AF, HR: 3.440; 95% CI: 1.828–6.472, *p* < 0.001; for Creatinine, HR: 1.011; 95% CI: 1.003–1.019, *p* = 0.011; for LVEF, HR:0.963; 95% CI: 0.964–0.994, *p* = 0.021; the myectomy group vs. the medical treatment group, HR,0.294; 95% CI: 0.125,0.690; *p* = 0.005). However, in multivariate analysis, there was no significant difference between the ASA group and the medical treatment group (the ASA group vs. the medical treatment group; HR, 0.910, 95% CI: 0.374, 2.213; *p* = 0.835).

**Table 4:** Predictors of all-cause mortality and cardiac transplantation.

**Parameter**	**Univariate cox analysis**		**Multivariate cox analysis**	
	**HR (95% CI)**	***p*-value**	**HR (95% CI)**	***p*-value**
Age	1.089 (1.063,1.116)	<0.001	1.051 (1.022,1.080)	<0.001
Male	0.799 (0.455,1.402)	0.434	–	–
Previous AF	4.450 (2.513,7.878)	<0.001	3.440 (1.828,6.472)	<0.001
Coronary artery disease	1.101 (0.534,2.271)	0.794	–	–
Cr (μmol/L)	1.020 (1.013,1.028)	<0.001	1.011 (1.003,1.019)	0.011
Baseline septal thickness, mm	0.980 (0.927,1.036)	0.481	–	–
LV ejection fraction (%)	0.95 (0.931,0.968)	<0.001	0.963 (0.933,0.994)	0.021
LV end-diastolic diameter (mm)	1.068 (1.031,1.108)	<0.001	1.014 (0.964,1.066)	0.592
Myectomy group vs. Medical treatment group	0.211 (0.104,0.429)	<0.001	0.294 (0.125,0.690)	0.005
ASA group vs. Medical treatment group	0.383 (0.169,0.867)	0.021	0.910 (0.374,2.213)	0.835

**Figure 2:**
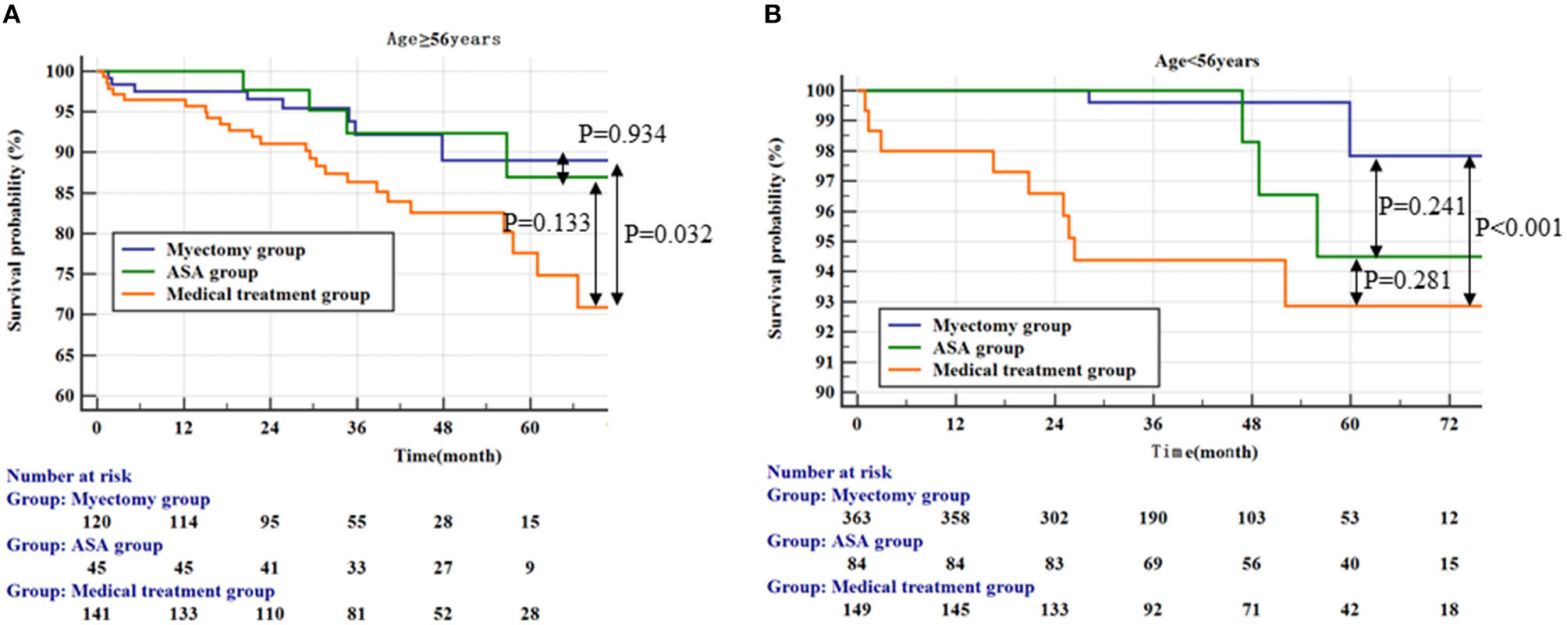
Kaplan–Meier curves comparing the probability of outcomes stratified by treatment strategies. **(A)** Represents the population ≥56 years; **(B)** Represents the population <56 years.

We conducted an ROC analysis on age factors. According to the analysis results, we found that 56 years old was the cut-off value (Supplementary Figure 3). Based on the cut-off value of 56 years old, we did a K-M survival analysis and a multivariate cox analysis. In the age ≥ 56 years group, there was no significant difference between the myectomy group and the ASA group (Figure 2A; the ASA group vs. the medical treatment group, *p* = 0.133 by log-rank test). But, compared with the drug treatment group, only the myectomy group was statistically significant compared with the medical treatment (Figure 2A; vs. the medical treatment group, *p* = 0.032 by log-rank test; vs. the ASA group, *p* = 0.934 by log-rank test). In the age <56 years group, compared with the medical treatment group, there was only still the myectomy group, which was statistically significant compared with the medical treatment (Figure 2B; vs. the medical treatment group, *p* < 0.001 by log-rank test). In addition, although there was still no statistical difference between the myectomy group and the ASA group, the ASA group was graphically better.

Two multivariate models were constructed in the age <56 years group and the age ≥ 56 years group, respectively (Table 5). In the multivariate cox regression analysis model 1 (age <56 years group), LVEF and myectomy were shown to be significantly associated with an all-cause mortality and cardiac transplantation (for LVEF, HR, 0.920; 95% CI: 0.893, 0.9623, *p* < 0.001; for the myectomy group vs. the medical treatment group, HR, 0.153; 95% CI: 0.031, 0.747, *p* < 0.02). In multivariate cox regression analysis model 2 (the age ≥ 56 years group), age was significantly associated with the endpoint (HR, 1.095; 95% CI: 1.040, 1.154; *p* < 0.001). Myectomy and ASA had no significant predictive value for an all-cause mortality and the cardiac transplantation endpoint in Model 2.

**Table 5:** Multivariate Cox Analysis for All-Cause Mortality and Cardiac transplantation respectively in two groups (age <56 year and age ≥56 year).

	**Model 1 (age** **<56 years group)**		**Model 2 (age** **≥56 years group)**	
	**HR (95% CI)**	***p*-value**	**HR (95% CI)**	***p*-value**
Age	1.003 (0.936,1.075)	0.931	1.084 (1.027,1.143)	0.003
Male	1.663 (0.320,8.651)	0.546	1.711 (0.850,3.442)	0.132
Previous AF	7.729 (2.248,26.574)	0.001	2.094 (1.000,4.384)	0.05
Baseline septal thickness, mm	1.054 (0.985,1.128)	0.127	0.993 (0.923,1.070)	0.862
LV ejection fraction (%)	0.918 (0.890,0.947)	<0.001	0.980 (0.948,1.014)	0.251
Myectomy group vs. Medical treatment group	0.140 (0.029,0.673)	0.014	0.558 (0.207,1.501)	0.248
ASA group vs. Without Medical treatment group	0.907 (0.221,3.724)	0.892	0.819 (0.265,2.529)	0.729

## Discussion

In this long-term survival study, we compared the prognosis of patients with HOCM after ASA, myectomy, and simple drug therapy. Among the three groups, the myectomy group had lower all-cause mortality rates and cardiac transplantation endpoint and a lower complication rate than ASA. Significant differences were also detected for age, previous AF, Creatinine, LV ejection fraction, LV end-diastolic diameter, and other influential factors in the three groups. In multivariate cox regression analysis, age, previous AF, Creatinine, LV ejection fraction, and treatment strategies were significantly associated with the endpoint. Specifically, in the overall population, the myectomy group and the ASA group all have significant statistical differences compared with the medical treatment group; although the surgical group was graphically better than the ASA group, there was no significant difference between the two groups. This conclusion is consistent with a previous study (18). Furthermore, according to the ROC curve of age, we selected the cut-off value of 56 years old for subgroup analysis. In the subgroup analysis, although there was no statistical difference between the myectomy group and the ASA group, compared with the medical treatment group, only the myectomy group had statistical differences in all subgroups.

By further adjusting for confounding factors, we found that myectomy was a significant protective factor only in the patients with age <56 years in the multivariate cox regression analysis. In the patients over 56 years old group, ASA and myectomy treatment had no significant difference in prognosis compared with the drug treatment group. In fact, in real clinical work, from the perspective of surgical safety, the treatment team generally recommends patients with younger grades to receive invasive treatment. Our retrospective study may provide some support for this view.

The rate of severe periprocedural complications (death, ventricular fibrillation/sustained VT, and ICD/Pacemaker) was close to the previous studies (11, 19, 20). Specifically, the rate of arrhythmia and the need for pacemaker or ICD installation during the perioperative period of ASA were higher than those in the myectomy group (Table 2). Generally, it shows that there is a greater need for pacemaker implantation after ASA (8, 9). The baseline age of patients with ASA is higher, which may confuse the relationship between patients with ASA, and the need for pacemaker implantation. Another explanation for the comparability of pacemaker implantation rates may be the wise use of alcohol in this study (21, 22). Absolute alcohol is injected into the interventricular septal branch through the interventional catheter. In fact, there are uncertain factors in this treatment method. For example, the branches of interventricular septal vessels are different in each patient, and the range of myocardial necrosis caused by absolute alcohol cannot be accurately controlled. At follow-up, the LVOT gradient in the patients with ASA was evidently higher than that in the patients with myectomy in this study. The recurrence rate of left ventricular outflow tract obstruction after ASA was also higher than that in the myectomy group; eight patients were treated by alcohol ablation before a definitive treatment by surgical myectomy. An alarming long-term complication after ASA is the ventricular arrhythmia, which may be induced after a ventricular septal infarction (5). In a single-center study, the incidence of ICD shock and SCD after ASA was higher than that after myectomy (13).

### Study Limitations and Strengths

This study was a nonrandomized, observational study. In particular, the average age of the three groups is different, which shows that selection bias plays a significant role. Similarly, there is a difference in the incidence of perioperative complications between ASA and myectomy. The strength of the study is the extended follow-up and the completeness of the data. An extensive search of all hospital records and operation reports was performed and was completed for complications in all patients. Questionnaires and, when necessary, consulting by telephone, were used to obtain a more objective result of the symptomatic status of the patient at a late-term follow-up. Finally, the advantage of this study is the analysis of perioperative complications and long-term outcomes.

## Conclusions

Our study further complements the survey on the choice of treatment strategies for HOCM (8–12). The patients with medical treatment seemed to suffer from the highest risk of achieving an all-cause mortality and the endpoint of heart transplantation. After ASA, the long-term survival and the clinical outcome are comparable to myectomy, but myectomy seemed better than ASA in the younger patients. Some disadvantages of percutaneous procedures have also been shown. Due to the less controllable degree, periprocedural complication frequency after ASA was higher than myectomy.

Furthermore, gradients after myectomy are lower at late follow-up. In addition, some conditions warrant a surgical approach, such as coronary septal anatomy unsuitable for ASA (23), such as (sub)valvular abnormalities, or multivessel coronary artery disease. To sum up, when selecting treatment strategies, the patients should be individually evaluated by a multidisciplinary team of cardiologists and surgeons.

## Data Availability Statement

The data analyzed in this study is subject to the following licenses/restrictions: All patients in this study were evaluated at the Fuwai Hospital (National Center of Cardiovascular Diseases, China). Requests to access these datasets should be directed to drtangyida@126.com.

## Ethics Statement

The studies involving human participants were reviewed and approved by the Ethics Committee of the Fuwai Hospital. The patients/participants provided their written informed consent to participate in this study.

## Author Contributions

Y-DT, W-yW, and XM: study concept and design. Y-DT, XM, W-yW, KZ, JG, and JZ: acquisition, analysis, and interpretation of data. XM, W-yW, KZ, CS, and Y-DT: drafting of the manuscript. KZ: English language editing. XM, W-yW, YL, and J-jW: statistical analysis. Y-DT: obtained funding and study supervision. All authors: critical revision of the manuscript for important intellectual content.

## Funding

This work was supported by the National Key Research and Development Program of China (2020YFC2004700), National Natural Science Foundation of China (81800327, 81900272, 81825003, and 91957123), Beijing Municipal Commission of Science and Technology (Z181100006318005), the Chinese Academy of Medical Sciences Innovation Fund for Medical Sciences (CIFMS 2016-I2M-1-009), and Project of Henan Medical Science and Technology Research Program 2019 (LHGJ20190781).

## Conflict of Interest

The authors declare that the research was conducted in the absence of any commercial or financial relationships that could be construed as a potential conflict of interest.

## Publisher's Note

All claims expressed in this article are solely those of the authors and do not necessarily represent those of their affiliated organizations, or those of the publisher, the editors and the reviewers. Any product that may be evaluated in this article, or claim that may be made by its manufacturer, is not guaranteed or endorsed by the publisher.
