# Supplementary figures and images for: Hypertrophic Obstructive Cardiomyopathy: Comparison of Outcomes After Myectomy or Alcohol Ablation
Source: Front Cardiovasc Med. 2022 Mar 14;9:755376. doi: 10.3389/fcvm.2022.755376 (PMC8964041; doi:10.3389/fcvm.2022.755376)

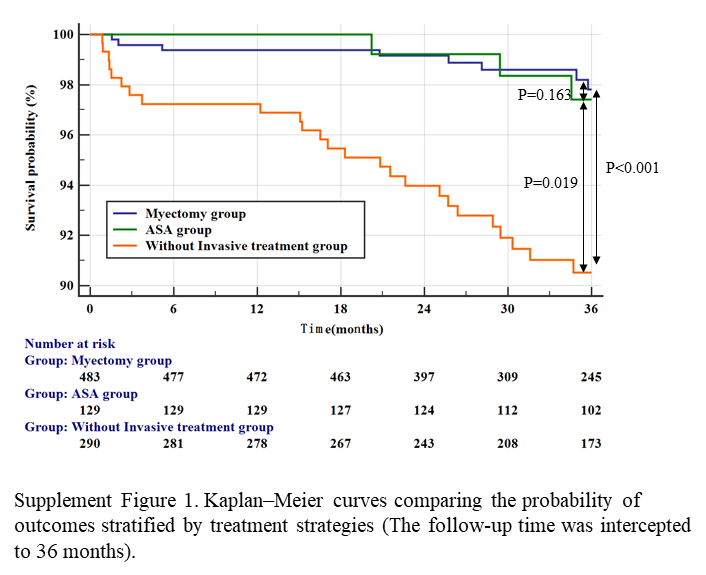

Supplement: Supplementary file 1 [file Image_1.TIF]

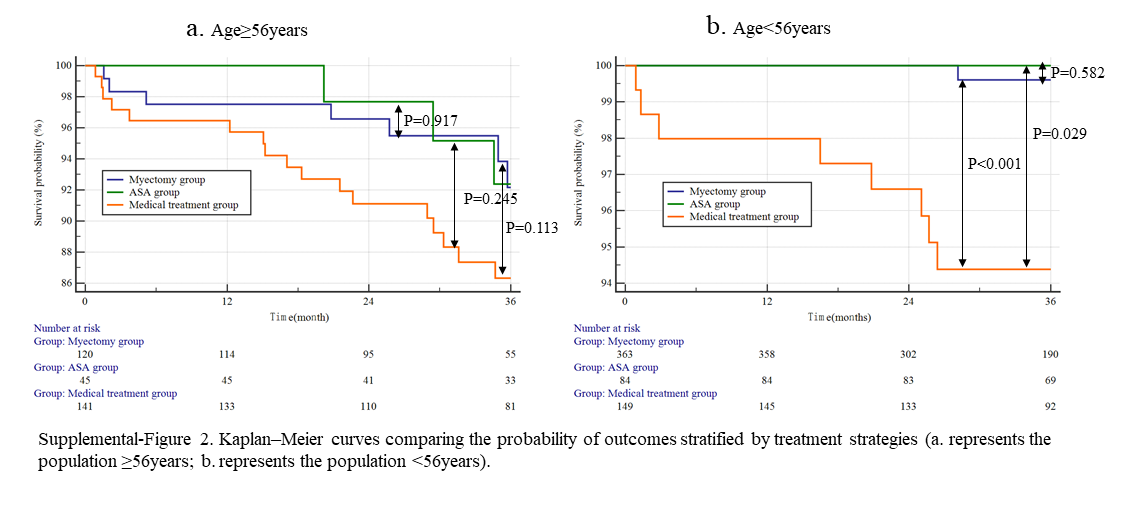

Supplement: Supplementary file 2 [file Image_2.TIF]

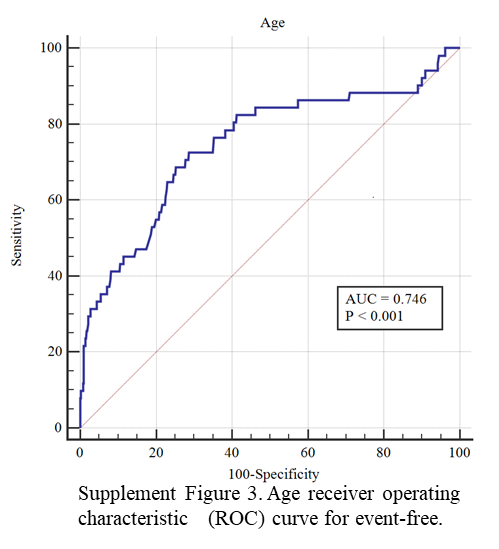

Supplement: Supplementary file 3 [file Image_3.TIF]
